# Home environment and noise disturbance in a national sample of multi-family buildings in Sweden-associations with medical symptoms

**DOI:** 10.1186/s12889-021-12069-w

**Published:** 2021-11-03

**Authors:** Juan Wang, Dan Norbäck

**Affiliations:** Department of Medical Sciences, Occupational and Environmental Medicine, Uppsala University, SE-751 85 Uppsala, Sweden

**Keywords:** Noise disturbance, Noise annoyance, Medical symptoms, Building factors, Renting, Urbanization, Traffic noise

## Abstract

**Background:**

Poor acoustic conditions at home can have negative health impact. The aim was to investigate home environment factors and medical symptoms associated with noise disturbance.

**Methods:**

All adults (≥18 y) registered in selected apartments in Sweden were invited to participate in a questionnaire survey including medical questions and personal factors. Totally 5775 adults participated (response rate 46%). Information on home environment was obtained through an indoor environment questionnaire. Two-level logistic regression models (individual, municipality) were performed to estimate associations.

**Results:**

Totally 11.9% reported noise disturbance in general at home. Noise disturbance from voice/radio/TV/music/similar sounds from neighbours (13.2%), scraping sound/footsteps/thumping from neighbours (16.5%) and road traffic (16.1%) were common. Younger age and smoking were related to more noise disturbance and more medical symptoms. Noise disturbance was related to tiredness, headache and difficulty concentrating (OR = 1.70–8.19). Renting the apartment (OR = 2.53) and living above ground floor (OR = 1.37) were related to more noise disturbance in general. Living in newer buildings (constructed from 1986 to 2005) was related to less noise disturbance in general (OR = 0.40–0.59). A warmer climate (OR = 1.95), higher municipality population density (OR = 1.24), a longer living time (OR = 1.34), construction year (1961–1975) (OR = 2.42), renting (OR = 1.80–2.32), living above ground floor (OR = 1.45) and having a bathroom fan (OR = 1.84) were associated with increased noise disturbance from neighbours. Factors associated with increased noise disturbance from installations or ventilation/fans/heat pumps included a warmer climate, higher municipality population density, construction year (1961–1995), renting and any mechanical ventilation. Higher municipality population density, construction year (especially 1961–1985) and renting were associated with more noise disturbance from traffic (OR = 1.77–3.92). Renting the apartment (OR = 1.73) and living above ground floor (OR = 1.60) were related to more severe traffic noise disturbances. Noise disturbance in general was partly a mediator of the effects of old buildings, renting the apartment and lack of mechanical ventilation on medical symptoms (% of total effect mediated by noise disturbance: 19–44.8%).

**Conclusions:**

Noise disturbance can be associated medical symptoms. Younger age, smoking, a warmer climate, higher municipality population density and different building factors (e.g. renting the apartment, construction period 1961–1985) can be associated with noise disturbance.

**Supplementary Information:**

The online version contains supplementary material available at 10.1186/s12889-021-12069-w.

## Background

Noise pollution, along with air pollution and water pollution, are considered to be three major forms of environmental pollution [1]. Environmental noise pollution corresponds to noise caused by road, rail and airport traffic, industry, construction, as well as some other outdoor activities. Noise can cause both auditory and non-auditory health effects [2]. Auditory health effects include tinnitus and noise induced hearing loss [2, 3]. Non-auditory health effects can include sleep disturbances, mental disturbances (stress, mood changes), physical effects (fatigability, headaches), cognitive and learning disorders, cardiovascular effects, etc. [2]. The mechanisms of the non-auditory noise-induced cardiovascular and metabolic consequences were discussed in one recent review [4].

There are international guidelines aiming to protect humans from harmful exposure to environmental noise. The World Health Organization (WHO) Guidelines for Community Noise 1999 recommend less than 30 A-weighted decibels (dBA) in bedrooms during the night for good sleep quality and less than 35 dBA in classrooms to allow good teaching and learning conditions [5]. Levels of 55 dBA is the current WHO guideline for acceptable outdoor noise levels at the most exposed façade of a building [5]. It is estimated that in Europe, 125 million people are exposed to road traffic noise greater than 55 dB (dBA) L_den_, 8 million are exposed to rail traffic noise and 3 million to aircraft noise above 55 dB (dBA) L_den_ (L_den_ is the common EU indicator that corresponds to the average noise level throughout the day, evening and night, to which a citizen is exposed over the period of a year) [6]. Almost 2 million people in Sweden are exposed to average noise levels exceeding national guideline value for outdoor noise (55 dBA) [7].

Noise annoyance, a commonly used marker of noise exposure, can be described as stress reaction involving individual physiological, emotional, cognitive, and behavioural responses [5, 8]. Standard questions on the degree of noise annoyance have been developed by the International Commission on the Biological Effects of Noise and the International Organization for Standardization, using an 11-point numerical scale and a 5-point semantic scale [9, 10]. Activities such as communication, relaxation/resting/sleeping as well as reading and intellectual work can be especially sensitive to noise disturbances [11].

Recent reviews indicated comprehensive evidence on road traffic noise exposure and increased risk of ischemic heart diseases and hypertension [12–14]. The WHO report “Burden of disease from environmental noise, 2011” describes that sleep disturbance and annoyance comprise the main burden of environmental noise: one in five individuals has disturbed sleep at night and one in three individuals is annoyed during the daytime because of traffic noise [1]. Annoyance was estimated to be the second major health effect of environmental noise after sleep disturbance, with more than 650 thousands healthy life years lost every year [1].

Research to date has focused on outdoor environmental noise (i.e. road traffic and aircraft noise), while little attention has been paid to noise from indoor sources. The predominant source of noise annoyance in residential quarters is traffic followed by neighbours. Neighbourhood noises usually include noise of footsteps, sounds with high information content such as language or music, etc. Most published studies focus on traffic noise caused health problems. Few studies exist on effects of neighbourhood noise on annoyance or health [15, 16]. One study from Sweden reported that the residents were twice more often annoyed from noise from installation of ventilation and air-conditioning systems than noise from traffic [16]. One recent study from UK showed that perceived neighbour noise level (talking/shouting and TV/music activities) increased substantially during the COVID-19 lockdown [17]. One large European study found that neighbourhood noise increased health risk for the cardiovascular system and increased risk of depression and migraine among adults [15].

More home environment studies are needed to investigate how noise can affect occupants’ health. Our study is part of the Building Energy, Technical Status and Indoor Environment (BETSI) study which consists of a stratified random sample of all multi-family buildings in Sweden. The first aim of our study was to estimate the prevalence of noise disturbance (traffic noise and neighbourhood noise) and medical symptoms. The second aim was to study associations between home environment factors and noise disturbance. The third aim was to study associations between noise disturbance and medical symptoms. Effects of exposure in the home environment on medical symptoms can be mediated by noise as well as other building-related factors. Mediation analysis is a statistical method that can be used to determine how much of the medical effects of a specific building factor that are mediated by noise disturbance. The fourth aim was to apply mediation analysis in this study.

## Methods

### BETSI study

The Swedish National Board of Housing, Building and Planning commissioned the BETSI (buildings, energy use, technical status and indoor environment) study in 2006. The BETSI study was aiming to obtain representative information of the status of Swedish buildings, as well as indoor environment in relation to occupants’ health. The selection of buildings was conducted by Statistics Sweden (SCB) by using a multi-stage sampling procedure [18]. Totally 30 Swedish municipalities were selected from a total of 290 municipalities across Sweden through a stratified random selection procedure, taking into account geographic and demographic characteristics (temperature zone and region), and degree of urbanization. Data on all buildings and their construction year from these 30 municipalities were obtained from the central building register in Sweden. Stratified random sampling was applied to sample buildings based on the construction year in five classes (before 1960, 1960–1975, 1976–1985, 1986–1995 and 1996–2005) aiming to get the same number of buildings in each age class. This leads to an over-sampling of new buildings, since most buildings in Sweden are old. The age classes were selected to reflect changes in the building codes.

### Study population and questionnaires

The present study consisted a subsample of 690 multi-family buildings (including 8841 apartments). Adults (≥18 years old) living in each apartment were identified by SCB from the Swedish civil registration register. All adults (≥18 years) registered in the selected apartments received a personal questionnaire including medical questions and personal factors. Moreover, one indoor environment questionnaire was sent to each apartment. The questionnaires were developed at Department of Medical Science, Uppsala University, based on previous studies [19–24]. The main focus was on respiratory and non-respiratory health, but one part of the questionnaire was about noise disturbance. The postal questionnaire was administered by SCB in the spring of 2008. Two reminders were sent to those who did not reply the first time. Totally 5775 adults participated in the current study and returned the personal questionnaire (46%), and a total of 4369 indoor environment questionnaires (49%) were returned.

### Questions on self-reported medical symptoms

One question included in the personal questionnaire asked: In the last 3 months, have you had any of the following symptoms? This question was followed by a list of symptoms. The current study included three general symptoms that could be influenced by stress: (1) tiredness (2); headache and (3) difficulty concentrating. There were three alternatives for each symptom: yes, often (every week); yes, sometimes; no, never. Those questions originated from the MM-questionnaire from Örebro University Hospital in Sweden [23]. Moreover, gender, age, current smoking habit and average time spends away during weekdays (0–4 h/5–9 h/10 h or more per day) were asked in the personal questionnaire.

### Questions on noise disturbance

In the personal questionnaire, there were five questions about noise disturbance at home:

1. How is the sound or noise condition in general at home? There were five alternatives: very good; good; acceptable; bad; very bad.
2. How much have you been disturbed by sound/noise in the last 3 months from sources inside the building, from: a) lines and pipes; b) ventilation/fans inside; c) voice/radio/TV/music/similar sounds from neighbours; d) scraping sound/footsteps/thumping/similar sounds from neighbours; e) amusement centre in the property; f) stairwell or elevators. There were six alternatives for each sub-question: not at all; slightly; moderately; very; extremely; not existed.
3. How much have you been disturbed by sound/noise in the last 3 months from sources outside the building, from: a) ventilation/fans/heat pumps; b) road traffic; c) train traffic; d) flight traffic. There were six alternatives for each sub-question: not at all; slightly; moderately; very; extremely; not existed.
4. Does traffic noise (road, train or flight traffic) lead to some of the following disturbances: a) difficult to hear radio/TV; b) telephone calls being affected; c) normal conversations being affected; d) rest/relaxation being disturbed; e) difficulties in sleeping; f) being woken up from traffic noise. There were three alternatives for each sub-question: yes, often (every week); yes, sometimes; never.

The variable “noise disturbance in general at home” was created based on question (1)], coded as 1 if the answer was “bad” or “very bad”, and coded as 0 if the answer was “very good”, “good” or “acceptable”.

For noise disturbance from each specific indoor source (question (2)) or outdoor source (question (3)), the answer “moderately”, “very” and “extremely” were coded as 1, and “not at all” and “slightly” were coded as 0.

For each traffic noise caused disturbance (question (4)), “yes, often (every week)” was coded as 1 and “yes, sometimes” and “never” were coded as 0. The variable “any severe traffic noise disturbance” was created, coded as 1 if the answer to any specific sub-question (question (4)) was coded as 1, and coded as 0 if the answer to all specific sub-questions were coded as 0.

Our questionnaire was in Swedish. We have used the term "noise disturbance" in our translation of the noise questions, but the term "noise annoyance" could also be a suitable description of the noise disturbance reactions reported in questions (2), (3) and (4).

### Questions on home environment

Data on home environment factors were gathered from the home environment questionnaire, including:

1. How long time have you been living in the current apartment (0–5 years; more than 5 years);
2. Total number of persons (children or adults) living at home;
3. Size of the home (m^2^ floor area);
4. Ownership of the current apartment (self-owned; renting);
5. Location of the apartment in the building (at ground floor/basement; above ground floor);
6. Any mechanical ventilation in the apartment (yes; no);
7. Bathroom fan (yes; no);
8. Window opening frequency (every day; less often).

A variable “crowdedness” was calculated based on total number of persons at home and size of the home (persons/100 m^2^).

Data on building construction year was collected from the National Building Register, Real Property Register and were categorized into five groups: before 1960, 1961–1975, 1976–1985, 1986–1995 and 1996–2005. Data on population and area of each municipality were obtained from wikipedia website [25]. Municipality population density (number of persons per km^2^) was calculated based on population and area of each municipality. Data on temperature zone were obtained from a Swedish government report [26]. Furthermore, municipality population density was divided to four quartiles: Quartile 1 ≤ 25%; 25% < Quartile 2 ≤ 50%; 50% < Quartile 3 ≤ 75%; 75% < Quartile 4 ≤ 100%. Temperature zone of each municipality was coded as: 1, inner part of north Sweden (Norrland); 2, costal part of Norrland and some inner part of Svealand; 3, Svealand (main parts); 4, Götaland (main parts). A higher number indicates a warmer climate.

### Statistical analysis

Chi-square test was applied to compare the prevalence of noise disturbance sources and stress-related symptoms between subgroups (stratified for gender, age and smoking). Initially, correlation analysis (Spearman correlation) was performed for 11 noise questions, including noise disturbance in general at home (one variable), noise from inside of the building (six variables) and noise from outside of the building (four variables). Similar correlation analysis was applied for home environment factors and for three medical symptoms. Two-level logistic regression models (individual, municipality) were performed to estimate associations between indoor and outdoor noise disturbance sources and medical symptoms, adjusting for gender, age and smoking (three covariates). Similar two-level models were applied to estimate associations between home environment factors and different noise disturbance sources, adjusting for the three covariates. Furthermore, similar two-level models were applied to estimate associations between home environment factors and medical symptoms, adjusting for the three covariates. As a next step, mutual adjustment logistic regression models (two-level) were constructed including all exposure variables (home environment factors) with *p* < 0.2 in single exposure models. Moreover, mediation analyses were performed for selected home environment factors in relation to medical symptoms, keeping noise disturbance in general at home as a mediator in the model. The statistic models used for mediation analyses of noise disturbance in general at home included: a) logistic regression models for each home environment factor in relation to noise disturbance in general at home; and b) logistic regression models for each home environment factor in relation to medical symptom, keeping noise disturbance in general at home in the model and adjusting for the three covariates.

The statistical analysis was conducted with Stata 15.1 (Stata Corp, Texas, USA) and SPSS 25.0 (SPSS Inc., Chicago, IL, USA). Associations were expressed as odds ratios (OR) with a 95% confidence interval (CI) for ordinal regression and logistic regression models. The medeff command (in Stata) was used for mediation analysis. In all statistical analyses, two-tailed tests and a 5% level of significance were applied.

## Results

A total of 5775 personal (46%) and 4369 indoor environment (49%) questionnaires were returned. Among the returned personal questionnaires, 73 had no information on gender, and 49 had no information on age. The comparison between participants and non-participants was performed by SCB Sweden [27] to check the representativeness of the questionnaire data. In this comparison, SCB linked demographic register data to participants and non-participants by using the national ID-number for each subject. There was no major difference in participation rate between different municipalities, between those being born in Sweden and foreign-born persons, and between Swedish citizens or non-Swedish citizens. Most of the differences in participation rate were small, except for age and civil status. Older persons and married persons had a higher participation rate. Among the participants: 56.5% were females; 63.5% aged between 18 to 65 years old, 36.5% were older than 65 years old; 12.0% were current smokers. A total of 40.1% of the participants spent 0–4 h away per day from home during weekdays, 39.0% spent 5–9 h and 20.0% were away 10 h or more.

Data on general noise disturbance is shown in Table 1. Totally 8.2% reported the sound or noise condition at home as “bad” and 3.7% reported it as “very bad”. Regarding noise disturbance from specific indoor or outdoor sources: few reported “very” and “extremely”, and slightly higher proportions reported “moderately”.

**Table 1:** Prevalence of noise disturbance

Noise disturbance	%	%	%	%	%
**Sound or noise condition in general at home**	Very good	Good	Acceptable	Bad	Very bad
	20.7	39.3	28.2	8.2	3.7
**Noise from inside of the building, from**	Not at all	Slightly	Moderately	Very	Extremely
Lines and pipes	59.6	31.6	5.6	2.2	1.0
Ventilation/fans inside	64.7	27.8	4.6	1.9	1.1
Voice, radio, TV, music or similar sounds from neighbors	49.5	37.3	7.4	3.1	2.7
Scraping sound, footsteps, thumping or similar sounds from neighbors	43.5	40.0	9.6	4.1	2.9
Amusement centre in the property	91.7	6.2	1.0	0.6	0.5
Stairwell, elevators	58.2	31.3	6.6	2.4	1.4
**Noise from outside of the building, from**	Not at all	Slightly	Moderately	Very	Extremely
Ventilation/fans/warm pumps	79.5	16.1	2.7	1.1	0.8
Road traffic	52.4	31.5	9.3	3.8	3.1
Train traffic	86.4	10.4	1.9	0.6	0.6
Flight traffic	83.8	12.9	2.1	0.6	0.6

Prevalence of noise disturbance and medical symptoms are shown in Table 2, stratified for gender, age and current smoking. Most commonly reported noise disturbance from inside the building were from voice/radio/TV/music/similar sounds from neighbours (13.2%) and scraping sound/footsteps/thumping from neighbours (16.5%). Road traffic was the most commonly reported noise disturbance from outdoor sources (16.1%). The most commonly reported traffic noise caused disturbances were being difficult to hear radio/TV (3.3%), rest/relaxation being affected (3.8%), difficulties in sleeping (3.3%) and being woken up from traffic noise (3.5%). Females reported more noise disturbance from voice/radio/TV/music/similar sounds from neighbours. Tiredness was the most common symptom (23.1%). Headache (8.5%) and difficulty concentrating (5.5%) were less common. Females had more symptoms than males (for all symptoms *p* < 0.001). In general, younger participants (≤ 65 y) and smokers reported more noise disturbance as well as more medical symptoms.

**Table 2:** Prevalence of noise disturbance and medical symptoms, stratified by gender, age and smoking

	Female *n* = 3219 ^a^ (%)	Male *n* = 2483 ^a^ (%)	*p* ^b^	18–40 y *n* = 1433 ^c^ (%)	41–65 y *n* = 2204 ^c^ (%)	> 65 y *n* = 2089 ^c^ (%)	*p* ^b^	Smoker *n* = 683 ^d^ (%)	Non-smoker *n* = 4993 ^d^ (%)	*p* ^b^	Total *n* = 5775 (%)
**Noise disturbance in general at home** ^**e**^	12.0	11.7	0.713	16.2	13.4	7.2	**< 0.001**	17.3	11.2	**< 0.001**	11.9
**Noise from inside of the building, from**											
Lines and pipes ^**f**^	8.9	8.7	0.771	12.9	9.8	4.9	**< 0.001**	11.8	8.4	**0.005**	8.9
Ventilation/fans inside ^**f**^	8.0	6.9	0.130	10.0	8.2	5.1	**< 0.001**	8.8	7.4	0.221	7.6
Voice, radio, TV, music or similar sounds from neighbors ^**f**^	14.1	12.0	**0.022**	21.6	13.6	6.8	**< 0.001**	14.9	13.0	0.169	13.2
Scraping sound, footsteps, thumping or similar sounds from neighbors ^**f**^	16.8	16.1	0.477	24.5	18.6	8.6	**< 0.001**	20.7	16.0	**0.003**	16.5
Amusement centre in the property ^**f**^	2.1	2.1	0.836	3.3	2.5	0.8	**< 0.001**	2.7	2.0	0.269	2.1
Stairwell, elevators ^**f**^	11.0	9.6	0.098	15.0	12.3	5.0	**< 0.001**	16.1	9.7	**< 0.001**	10.5
**Noise from outside of the building, from**											
Ventilation/fans/warm pumps ^**f**^	4.7	4.0	0.225	4.0	5.4	3.6	**0.011**	3.6	4.5	0.299	4.5
Road traffic ^**f**^	16.7	15.4	0.183	18.3	18.0	12.6	**< 0.001**	19.2	15.7	**0.021**	16.1
Train traffic ^**f**^	3.4	2.7	0.183	3.4	3.6	2.3	0.053	2.3	3.2	0.205	3.1
Flight traffic ^**f**^	3.4	3.1	0.575	3.5	4.0	2.4	**0.013**	2.9	3.4	0.508	3.3
**Any severe traffic noise disturbance** ^**g,h**^	7.5	6.7	0.267	8.1	7.9	5.5	**0.004**	8.0	7.0	0.387	7.2
**Medical symptoms**											
Weekly tiredness	26.3	19.0	**< 0.001**	31.7	22.6	16.7	**< 0.001**	27.0	22.6	**0.015**	23.1
Weekly headache	10.6	5.6	**< 0.001**	10.6	9.6	5.2	**< 0.001**	12.7	7.9	**< 0.001**	8.5
Weekly difficulty concentrating	6.5	4.1	**< 0.001**	7.2	5.6	3.8	**< 0.001**	6.1	5.4	0.462	5.5

Bold values indicate *p* < 0.05.

^a^Participants with missing data on gender (*n* = 73) were excluded

^b^*p* value by 2 × 3 Chi-square test

^c^Participants with missing data on age (*n* = 49) were excluded

^d^Participants with missing data on ownership (*n* = 99) were excluded

^e^The prevalence of reporting “bad” and “very bad” in the general noise disturbance question

^f^The prevalence of reporting “moderately”, “very” and “extremely” in the specific noise disturbance question

^g^Any severe traffic noise disturbance was defined as reporting “often” to any of the following traffic noise caused disturbances: difficult to hear radio/TV, telephone calls being affected, normal conversations being affected, rest/relaxation being affected, difficulties in sleeping and being woken up from traffic noise

^h^The prevalence of reporting “yes, often (every week)”

**Fig. 1:**
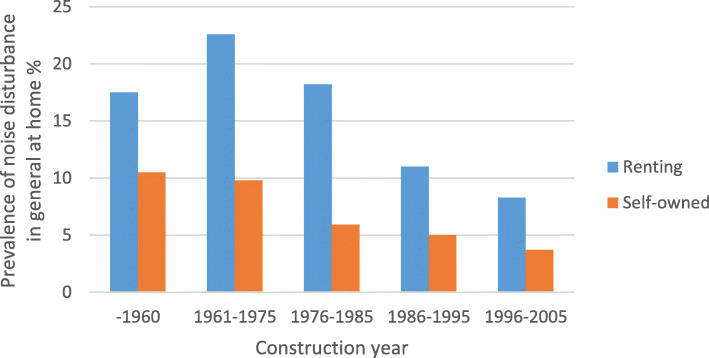
Prevalence of noise disturbance in general at home in different construction periods, stratified by ownership

**Fig. 2:**
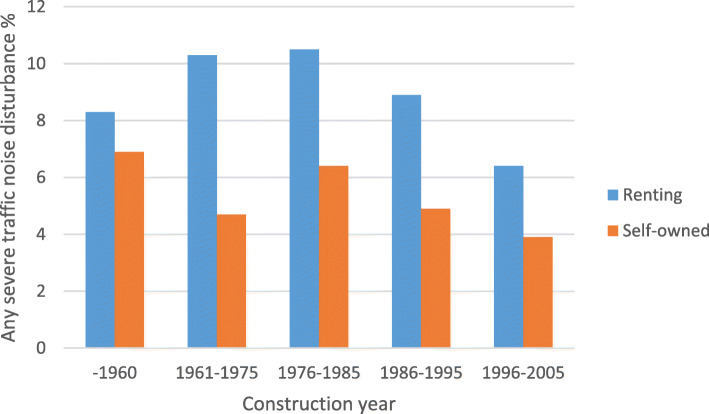
Prevalence of any severe traffic noise disturbance in different construction periods, stratified by ownership

**Fig. 3:**
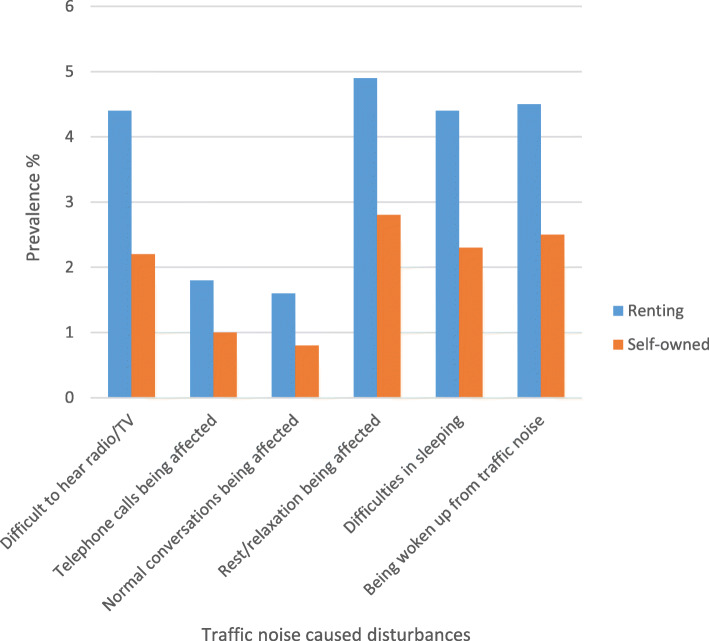
Prevalence of different traffic noise caused disturbances, stratified by ownership

**Fig. 4:**
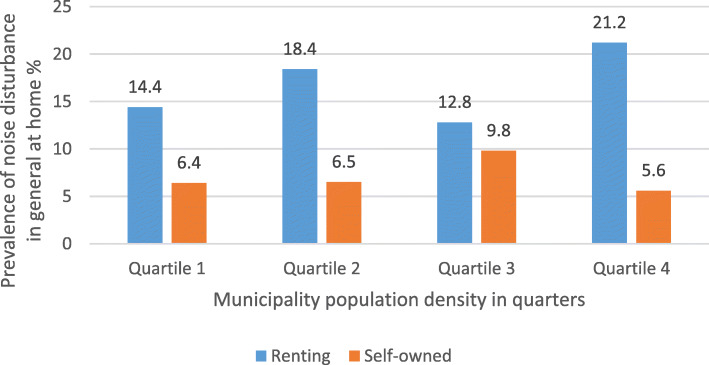
Prevalence of noise disturbance in general at home in relation to urbanization, stratified by ownership

**Fig. 5:**
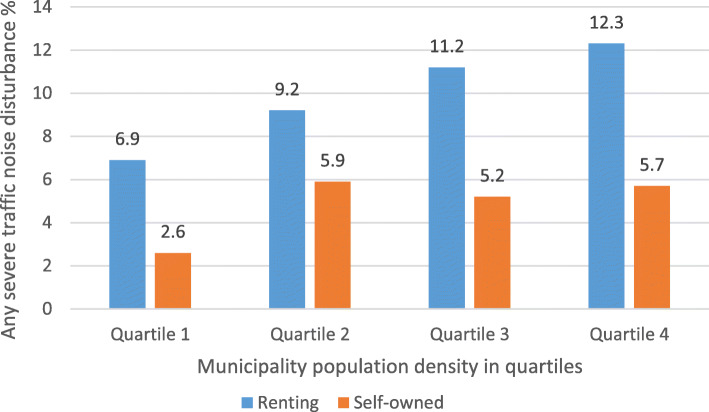
Prevalence of any severe traffic noise disturbance in different population quartiles, stratified by ownership

The prevalence noise disturbance in general at home and any severe traffic noise disturbance in different construction periods, stratified by ownership (self-owned/renting) are shown in Fig. 1 and Fig. 2, respectively. The prevalence of traffic noise related disturbances were shown in Fig. 3, stratified by ownership. Participants living in buildings constructed during 1961–1975 and 1976–1985 reported more noise disturbance (Fig. 1 and Fig. 2). Reported noise disturbance in general at home, traffic noise related disturbances and any severe traffic noise disturbance were all higher among those living in rented apartments as compared to those living in self-owned apartments (Fig. 1, Fig. 2 and Fig. 3). Increased municipality population density (indicated by four quartiles) was related to a higher prevalence of noise disturbance in general at home (Fig. 4) and a higher prevalence of any severe traffic noise disturbance (Fig. 5).

Associations between noise disturbance and medical symptoms are shown in Table 3. All types of noise disturbance were associated with tiredness, headache or difficulty concentrating.

**Table 3:** Associations between noise disturbance and medical symptoms OR (95%CI)^a^

Noise disturbance	Weekly tiredness	*p*	Weekly headache	*p*	Weekly difficulty concentrating	*p*
**Noise disturbance in general at home**	2.41(2.01,2.89)	**< 0.001**	3.74(2.96,4.72)	**< 0.001**	3.50(2.63,4.65)	**< 0.001**
**Noise from inside of the building, from**						
Lines and pipes	1.84(1.48,2.28)	**< 0.001**	2.81(2.13,3.71)	**< 0.001**	2.11(1.49,3.00)	**< 0.001**
Ventilation/fans inside	1.85(1.47,2.34)	**< 0.001**	2.44(1.80,3.32)	**< 0.001**	2.49(1.73,3.57)	**< 0.001**
Voice, radio, TV, music or similar sounds from neighbors	1.82(1.52,2.18)	**< 0.001**	2.90(2.28,3.69)	**< e0.001**	2.31(1.71,3.13)	**< 0.001**
Scraping sound, footsteps, thumping or similar sounds from neighbors	1.70(1.43,2.01)	**< 0.001**	2.76(2.19,3.47)	**< 0.001**	2.23(1.68,2.96)	**< 0.001**
Amusement centre in the property	1.42(0.93,2.17)	0.106	2.45(1.46,4.09)	**0.001**	2.39(1.30,4.38)	**0.005**
Stairwell, elevators	2.07(1.69,2.53)	**< 0.001**	3.52(2.73,4.54)	**< 0.001**	2.85(2.08,3.92)	**< 0.001**
**Noise from outside of the building, from**						
Ventilation/fans/warm pumps	1.96(1.47,2.60)	**< 0.001**	3.73(2.65,5.24)	**< 0.001**	3.26(2.17,4.90)	**< 0.001**
Road traffic	1.96(1.67,2.32)	**< 0.001**	2.44(1.95,3.07)	**< 0.001**	2.19(1.65,2.90)	**< 0.001**
Train traffic	1.87(1.33,2.61)	**< 0.001**	3.29(2.19,4.94)	**< 0.001**	2.88(1.78,4.69)	**< 0.001**
Flight traffic	2.16(1.56,2.97)	**< 0.001**	2.70(1.78,4.08)	**< 0.001**	2.96(1.84,4.75)	**< 0.001**
**Severe traffic noise disturbances such as**						
Difficult to hear radio/TV	3.23(2.36,4.41)	**< 0.001**	5.57(3.92,7.91)	**< 0.001**	5.29(3.55,7.89)	**< 0.001**
Telephone calls being affected	3.48(2.15,5.62)	**< 0.001**	7.10(4.22,11.9)	**< 0.001**	6.52(3.67,11.6)	**< 0.001**
Normal conversations being affected	3.74(2.25,6.22)	**< 0.001**	5.78(3.33,10.0)	**< 0.001**	7.11(3.97,12.7)	**< 0.001**
Rest/relaxation being affected	2.91(2.17,3.89)	**< 0.001**	5.24(3.77,7.28)	**< 0.001**	4.13(2.79,6.10)	**< 0.001**
Difficulties in sleeping	3.93(2.87,5.38)	**< 0.001**	8.19(5.81,11.6)	**< 0.001**	4.97(3.29,7.50)	**< 0.001**
Being woken up from traffic noise	3.75(2.77,5.09)	**< 0.001**	7.58(5.45,10.5)	**< 0.001**	5.79(3.93,8.53)	**< 0.001**
**Any severe traffic noise disturbance** ^**b**^	2.87(2.30,3.58)	**< 0.001**	5.02(3.85,6.55)	**< 0.001**	4.74(3.46,6.48)	**< 0.001**

Bold values indicate *p* < 0.05.

^a^Two-level logistic regression models (individual, municipality). The odds ratios were adjusted for gender, age and smoking

^b^Any severe traffic noise disturbance was defined as reporting “often” to any of the following traffic noise caused disturbances: difficult to hear radio/TV, telephone calls being affected, normal conversations being affected, rest/relaxation being affected, difficulties in sleeping and being woken up from traffic noise

The majority were living in warmer climate zones. The median value of municipality population density was 1179 person/km^2^ and the median value of crowdedness was 2.3 person/100m^2^. Over half (52.6%) of the participants had lived in the current apartment more than 5 years. Totally 41.6% of the participants were living alone and 40.1% reported that there were two persons living in the apartment. Totally 11.7% reported any children at home. About half (50.7%) of the participants had rented apartments. The majority were living above ground floor (81.8%). About half of the apartments had mechanical ventilation (45.4%) but only 14.6% had a bathroom fan. A total of 73.9% ventilated their homes on a daily basis by opening windows. Living in current apartment more than 5 years, construction year 1961–1975, renting and living above ground floor were all associated with noise disturbance in general at home. Living in newer buildings (constructed from 1986 to 2005) and having mechanical ventilation were related to less noise disturbance in general at home (Table S1 in Additional file 1).

Correlation analyses showed that correlation between different indoor and outdoor noise disturbance sources were low (Spearman correlation coefficient < 0.5), except the correlation between noise disturbance from voice/radio/TV/music/similar sounds and noise disturbance from scraping sound/footsteps/thumping/similar sounds (Spearman correlation coefficient = 0.57) (Table S2 in Additional file 1). The correlation coefficients between home environment factors were all low (Spearman correlation coefficient < 0.5) (Table S3 in Additional file 1). The correlation between average time spends away from home and noise disturbance in general at home was low (Spearman correlation coefficient = 0.08). The correlation between the three medical symptoms were all low (Spearman correlation coefficient < 0.5).

Three significant factors were identified in the mutual adjustment model including all variables with *p* < 0.2 in Table S1 (see Additional file 1): renting the apartment and living above ground floor were associated with more noise disturbance in general at home (Table 4). Living in newer buildings (constructed from 1986 to 2005) was associated with less noise disturbance in general at home (Table 4).

**Table 4:** Associations between home environment factors and noise disturbance in general at home OR (95%CI) ^a^

Home environment factors		Median (min, max)	%	Noise disturbance in general at home	*p*
Crowdedness ^b^		2.30(0.51,27.6)		1.04(0.95,1.15)	0.393
Total number of persons	1		41.6	0.85(0.66,1.08)	0.185
	2		40.1	0.88(0.60,1.29)	0.515
	3		9.8	0.79(0.48,1.31)	0.359
	4 or more		8.5		
Time living in current apartment	≤ 5 years		47.4	1.00	
	> 5 years		52.6	1.21(0.97,1.50)	0.085
Construction year	−1960		12.5	1.00	
	1961–1975		33.0	1.26(0.94,1.70)	0.126
	1976–1985		17.9	0.98(0.69,1.39)	0.899
	1986–1995		16.0	0.59(0.40,0.88)	**0.009**
	1996–2005		20.7	0.40(0.26,0.60)	**< 0.001**
Ownership	Self-owned		49.3	1.00	
	Renting		50.7	2.53(2.03,3.14)	**< 0.001**
Location of the apartment	Ground floor/basement		18.2	1.00	
	Above ground floor		81.8	1.37(1.05,1.80)	**0.023**
Any mechanical ventilation	Yes		54.6	0.84(0.68,1.03)	0.092
Bathroom fan	Yes		14.6	0.78(0.57,1.06)	0.112

Bold values indicate *p* < 0.05.

^a^Two-level logistic regression models (individual, municipality). Mutual adjustment including all variables with *p* < 0.2 in Table S1 (see Additional file 1). The odds ratios were adjusted for gender, age and smoking

^b^The ORs were expressed per 1 unit increase for crowdedness (person/100m^2^)

As a next step, associations between home environment factors and noise disturbance from specific indoor sources were analysed (see Table S4 and S5 in Additional file 1). Mutual adjustment models including all variables with *p* < 0.2 in Table S4 and S5 (see Additional file 1) are found in Tables 5 and 6. Renting the apartment was a consistent risk factor for noise disturbance from all six specific indoor sources. Higher municipality population density was related to noise disturbance from lines and pipes and amusement centre in the property. Living in a warmer climate was related to more noise disturbance from amusement centre in the property and from stairwell/elevators. Living in buildings constructed from 1961 to 1975 was associated with noise disturbance from lines and pipes and amusement centre in the property. Living in newer buildings (constructed from 1996 to 2005) was associated with less noise disturbance from voice/radio/TV/music/similar sounds and scraping sound/footsteps/thumping/similar sounds from neighbours. Moreover, living above ground floor, having mechanical ventilation and a bathroom fan were associated with more noise disturbance from indoor sources. Daily window opening was related to less noise disturbance from ventilation/fans inside the home.

**Table 5:** Associations between home environment factors and noise disturbance from specific indoor sources OR (95%CI) ^a^

Home environment factors		Lines and pipes	*p*	Ventilation/fans inside	*p*	Voice, radio, TV, music, similar sounds from neighbors	*p*
Municipality population density ^b^		1.07(1.01,1.14)	**0.020**	–	–	–	–
Time living in current apartment	≤ 5 years	–	–	–	–	1.00	
	> 5 years	–	–	–	–	1.23(0.99,1.52)	0.059
Construction year	−1960	1.00		1.00		1.00	
	1961–1975	1.86(1.27,2.74)	**0.002**	1.55(0.96,2.52)	0.076	1.28(0.96,1.72)	0.094
	1976–1985	1.21(0.77,1.90)	0.403	2.40(1.45,3.95)	**0.001**	0.88(0.63,1.24)	0.474
	1986–1995	1.33(0.84,2.11)	0.223	2.51(1.51,4.17)	**< 0.001**	0.62(0.43,0.91)	**0.014**
	1996–2005	1.02(0.65,1.61)	0.932	0.94(0.54,1.61)	0.813	0.34(0.22,0.51)	**< 0.001**
Ownership	Self-owned	1.00		1.00		1.00	
	Renting	1.88(1.47,2.39)	**< 0.001**	1.44(1.12,1.87)	**0.005**	2.07(1.69,2.55)	**< 0.001**
Location of the apartment	Ground floor/basement	1.00		–	–	1.00	
	Above ground floor	1.09(0.81,1.48)	0.555	–	–	1.45(1.10,1.90)	**0.008**
Any mechanical ventilation		0.82(0.65,1.04)	0.099	1.30(1.002,1.69)	**0.048**	1.01(0.82,1.24)	0.953
Bathroom fan	Yes	–	–	–	–	1.08(0.81,1.43)	0.604
Window opening frequency	Less often	–	–	1.00		–	–
	Everyday	–	–	0.70(0.54,0.90)	**0.006**	–	–

Bold values indicate *p* < 0.05.

^a^Two-level logistic regression models (individual, municipality). Mutual adjustment including all variables with *p* < 0.2 in Table S4 (see Additional file 1). The odds ratios were adjusted for gender, age and smoking

^b^The ORs were expressed per 1000 increase for municipality population density (number of persons per km^2^, person/km^2^)

**Table 6:** Associations between home environment factors and noise disturbance from specific indoor sources OR (95%CI) ^a^

Home environment factors		Scraping sound, footsteps, thumping or similar sounds from neighbours	*p*	Amusement centre in the property	*p*	Stairwell, elevators	*p*
Temperature zone ^b^		–	–	1.95(1.20,3.15)	**0.007**	1.21(1.04,1.41)	**0.016**
Municipality population density ^c^		–	–	1.24(1.03,1.49)	**0.022**	–	–
Time living in current apartment	≤ 5 years	1.00		–	–	1.00	
	> 5 years	1.34(1.10,1.62)	**0.003**	–	–	1.22(0.96,1.54)	0.103
Construction year	−1960	1.00		1.00		1.00	
	1961–1975	1.23(0.94,1.60)	0.137	2.42(1.25,4.67)	**0.008**	1.29(0.93,1.78)	0.132
	1976–1985	0.67(0.49,0.93)	**0.017**	0.88(0.38,2.03)	0.765	0.83(0.56,1.24)	0.365
	1986–1995	0.62(0.44,0.87)	**0.006**	1.32(0.58,3.01)	0.513	0.74(0.49,1.13)	0.165
	1996–2005	0.57(0.41,0.80)	**0.001**	0.66(0.27,1.61)	0.358	0.63(0.41,0.96)	**0.031**
Ownership	Self-owned	1.00		1.00		1.00	
	Renting	1.80(1.50,2.16)	**< 0.001**	2.32(1.49,3.63)	**< 0.001**	2.19(1.73,2.76)	**< 0.001**
Location of the apartment	Ground floor/basement	1.00		–	–	–	–
	Above ground floor	1.11(0.88,1.40)	0.387	–	–	–	–
Any mechanical ventilation		1.06(0.88,1.28)	0.506			0.89(0.71,1.11)	0.297
Bathroom fan	Yes	–	–	1.84(1.16,2.93)	**0.010**	–	–
Window opening frequency	Less often	–	–	–	–	–	–
	Everyday	–	–	–	–	–	–

Bold values indicate *p* < 0.05.

^a^Two-level logistic regression models (individual, municipality). Mutual adjustment including all variables with *p* < 0.2 in Table S5 (see Additional file 1). The odds ratios were adjusted for gender, age and smoking

^b^The ORs were expressed per 1 unit increase for temperature zone

^c^The ORs were expressed per 1000 increase for municipality population density (number of persons per km^2^, person/km^2^)

The associations between home environment factors and noise disturbance from specific outdoor sources and any severe traffic noise disturbance were analysed (see Table S6 in Additional file 1). Mutual adjustment model including all variables with *p* < 0.2 in Table S6 (see Additional file 1) are found in Table 7. Noise disturbance from outdoor sources were associated with various building factors, such as renting the apartment, certain construction period and window opening habit. Living in buildings constructed after 1960 was associated with more noise disturbance from train traffic. Living in newer buildings (constructed from 1996 to 2005) was related to less noise disturbance from flight traffic.

**Table 7:** Associations between home environment factors and noise disturbance from specific outdoor sources OR (95%CI)^a^

Home environment factors		Ventilation/fans/warm pumps	*p*	Road traffic	*p*	Train traffic	*p*	Flight traffic	*p*	Any severe traffic noise effect (often vs. less or never)	*p*
Temperature zone ^b^		1.22(0.98,1.53)	0.081	–	–	–	–	–	–	1.35(0.99,1.84)	0.059
Municipality population density ^c^		1.13(1.05,1.22)	**0.002**	–	–	1.42(1.02,1.99)	**0.039**	2.00(1.14,3.50)	**0.015**	–	–
Time living in current apartment	≤ 5 years	1.00		–	–	–	–	1.00		–	–
	> 5 years	0.93(0.67,1.28)	0.641	–	–	–	–	1.00(0.71,1.41)	0.989	–	–
Construction year	−1960	1.00		1.00		1.00		1.00		1.00	
	1961–1975	1.81(1.01,3.24)	**0.046**	0.92(0.70,1.21)	0.556	3.27(1.55,6.92)	**0.002**	1.57(0.91,2.72)	0.106	1.03(0.70,1.54)	0.868
	1976–1985	2.43(1.32,4.47)	**0.004**	0.75(0.54,1.02)	0.067	3.19(1.45,7.05)	**0.004**	2.35(1.32,4.17)	**0.004**	1.16(0.75,1.81)	0.499
	1986–1995	2.15(1.15,4.03)	**0.017**	0.77(0.56,1.06)	0.110	2.18(0.93,5.09)	0.073	1.36(0.74,2.49)	0.319	0.96(0.60,1.54)	0.867
	1996–2005	0.92(0.47,1.80)	0.807	0.81(0.59,1.09)	0.167	3.92(1.80,8.53)	**0.001**	0.39(0.18,0.84)	**0.016**	0.72(0.45,1.15)	0.175
Ownership	Self-owned	1.00		1.00		1.00		1.00		1.00	
	Renting	1.55(1.14,2.11)	**0.005**	1.77(1.48,2.13)	**< 0.001**	2.52(1.75,3.62)	**< 0.001**	1.28(0.91,1.78)	0.152	1.73(1.33,2.25)	**< 0.001**
Location of the apartment	Ground floor/basement	–	–	–	–	–	–	–	–	1.00	
	Above ground floor	–	–	–	–	–	–	–	–	1.60(1.11,2.29)	**0.011**
Any mechanical ventilation	Yes	1.46(1.06,2.00)	**0.020**	0.95(0.80,1.14)	0.596					0.92(0.71,1.19)	0.515
Bathroom fan	Yes	–	–	–	–	–	–	–	–	–	–
Window opening frequency	Less often	1.00		1.00		–	–	1.00		1.00	
	Everyday	0.64(0.47,0.88)	**0.005**	1.10(0.90,1.33)	0.355	–	–	1.56(1.05,2.31)	**0.026**	1.20(0.90,1.60)	0.208

Bold values indicate *p* < 0.05.

^a^Two-level logistic regression models (individual, municipality). Mutual adjustment including all variables with *p* < 0.2 in Table S6 (see Additional file 1). The odds ratios were adjusted for gender, age and smoking

^b^The ORs were expressed per 1 unit increase for temperature zone

^c^The ORs were expressed per 1000 increase for municipality population density (number of persons per km^2^, person/km^2^)

The associations between home environment factors and weekly medical symptoms are shown in Table S7 (see Additional file 1). Living in buildings constructed from 1996 to 2005 was associated with less medical symptoms. Renting was related to all types of symptoms. Having mechanical ventilation was related to less reporting of any symptoms and headache. Higher total number of persons living at home and having any children at home were both associated with headache. Having two or three persons living at home was associated with less tiredness, difficulty concentrating and any symptom. Having four or more persons at home was related to more headache. Living in the current apartment more than 5 years was associated with less tiredness.

Table 8 shows the associations between selected home environment factors and medical symptoms, as well as the mediation effects of noise disturbance in general at home on the associations. Significant mediation effects were found between old buildings (constructed before 1986), headache, difficulty concentrating and any symptom: the proportion of the total effect mediated by noise disturbance were between 33.3–44.8%. Significant mediation effects were found between renting the apartment and all types of symptoms (% of total effect mediated: 26.4–33.9%). Moreover, significant mediation effects were shown between lack of mechanical ventilation and headache (% of total effect mediated: 28.8%) and any symptom (% of total effect mediated: 19.0%).

**Table 8:** Associations between home environment factors and symptoms, and mediation effects of noise disturbance

Home environment factors		Any weekly symptom			Weekly tiredness			Weekly headache			Weekly difficulty concentrating		
		OR(95%CI) ^a^	*p*	%(95CI) ^b^	OR(95%CI) ^a^	p	%(95CI) ^b^	OR(95%CI) ^a^	*p*	%(95CI) ^b^	OR(95%CI) ^a^	*p*	%(95CI) ^b^
Construction year	New buildings ^c^	1.00			1.00			1.00			1.00		
	Old buildings ^d^	1.20(1.05,1.38)	**0.009**	**44.8(25.1208)**	1.11(0.96,1.27)	0.157	63.7(− 422,777)	1.44(1.15,1.80)	**0.001**	**39.2(24.3113)**	1.56(1.17,2.06)	**0.002**	**33.3(20.5,92.3)**
Ownership	Self-owned	1.00			1.00			1.00			1.00		
	Renting	1.44(1.26,1.65)	**< 0.001**	**27.1(19.4,45.1)**	1.33(1.16,1.52)	**< 0.001**	**33.9(22.4,70.5)**	1.94(1.56,2.41)	**< 0.001**	**26.4(19.6,39.6)**	1.70(1.30,2.22)	**< 0.001**	**30.0(19.6,61.6)**
Lack of mechanical ventilation	No	1.00			1.00			1.00			1.00		
	Yes	1.20(1.04,1.39)	**0.014**	**19.0(10.0,96.2)**	1.12(0.96,1.30)	0.136	29.0(−197,416)	1.34(1.06,1.68)	**0.015**	**28.8(15.9135)**	1.28(0.97,1.69)	0.086	27.8(− 139,226)

Bold values indicate *p* < 0.05.

^a^ Two-level logistic regression models (individual, municipality). The odds ratios were adjusted for gender, age and smoking

^b^ % of total effect mediated by noise disturbance in general at home

^c^ Constructed before 1986

^d^Constructed from 1986 to 2005

## Discussion

Noise disturbance in general at home and noise disturbance from indoor as well as from outdoor sources were common, and were associated with medical symptoms. We found that personal factors, climate, degree of urbanization as well as building related factors can influence noise disturbance. Younger age and current smoking were related to more noise disturbance. Living in a warmer climate and in areas with higher municipality population density (higher degree of urbanization) were associated with noise disturbance from indoor or outdoor sources. Renting the apartment was associated with noise disturbance from different indoor and outdoor sources. Noise disturbance was most common among those living in buildings constructed from 1961 to 1985. Living in newer buildings (constructed from 1986 to 2005) was associated with less noise disturbance. Apartment situated above ground floor was related to more noise disturbance in general and more traffic noise caused disturbances. Having mechanical ventilation was related to more noise disturbance from ventilation/fans/heat pumps and more traffic noise related disturbances. We found that noise disturbance in general at home was partly a mediator for the effects of old buildings, renting the apartment and lack of mechanical ventilation on medical symptoms.

### Prevalence of noise disturbance in the home

Reporting of noise disturbance in general at home was common in our study (11.9%). We found only one previous study from Sweden investigating the prevalence of noise annoyance from different sources among residents. The study suggested that complaints of annoyance to traffic noise (8.7%) and sound from neighbours (7.8%) were most common among participants, followed by sounds from ventilation systems (3.9%), sounds from other installations (2.4%) and industry noise (1.1%) [28]. The prevalence of noise disturbance from neighbours (voice/radio/TV/music/similar sounds (13.2%) and scraping sound/footsteps/thumping/similar sounds (16.5%)) found in our study were somewhat higher as compared to one previous Swedish study [28]. Reporting of noise disturbance from amusement centre in the property was less common in our study (2.1%).

A total of 8.9% reported noise disturbance from lines and pipes, and 10.5% reported noise disturbance from stairwell/elevators. The prevalence of noise disturbance from installations in our study was higher as compared to one previous Swedish study, which showed a prevalence of 2.4% for noise from installation [28]. Another study from Sweden suggested that the proportion of persons who were annoyed of noise from installations was more than twice as high as for traffic noise [16]. Installations, such as ventilation and air-conditioning systems, can generate low frequency noise (20–200 Hz) and may cause severe annoyance and sleep disturbance. Thus, it can be important to regulate the noise exposure from such installations.

We found that 7.6% were disturbed by noise from ventilation/fans inside the building, and 4.5% were disturbed by noise from ventilation/fans/heat pumps located outside the building. The prevalence of such disturbance found in our study was slightly higher than similar data from one previous Swedish study [28].

### Prevalence of noise disturbance from traffic

We found that reporting of noise disturbance from road traffic, train traffic and flight traffic were 16.1, 3.1 and 3.3%, respectively. Road traffic and railway noise contribute significantly to the burden of disease in Sweden each year (use disability-adjusted life-years (DALY) measure) [29]. The most important contributor to the noise related disease burden (DALY) was sleep disturbances (54%), followed by annoyance (30%) and cardiovascular diseases (16%) [29]. Aircraft noise can be the most annoying transportation noise when standardized for noise exposure level [5, 30, 31].

### Home environment factors associated with severe traffic noise disturbances

We found that renting the apartment was associated with severe traffic noise disturbances. Rented apartment buildings in Sweden are more likely to be located in poor areas such as major roads or train tracks. Living above ground floor was associated with severe traffic noise disturbances. There can be noise remedies such as concrete walls to be placed between buildings and major roads/train tracks to reduce traffic noise. This types of noise remedies can be effective to reduce noise levels for people living at ground floor or basement but less or not effective for people living above ground floor. Living at basement can be less disturbed by noise as small windows are often used in basement. Moreover, the ground connected to basement can help to reduce noise.

### Noise disturbance from indoor and outdoor sources associated with medical symptoms

Tiredness was the most commonly reported symptom in our study (23.1%), followed by headache (8.5%) and difficulty concentrating (5.5%). We found that noise disturbance in general at home, noise disturbance from indoor and outdoor sources, as well as reported severe traffic noise disturbances were all associated with all types of symptoms.

People annoyed by noise may experience a variety of negative responses, such as anger, disappointment, dissatisfaction, withdrawal, helplessness, depression, anxiety, distraction, agitation, or exhaustion [10, 32]. The symptoms included in our study can be caused by stress as well as other factors. Few studies have shown that noise annoyance and its relation with headache/tiredness. One experiment study from Sweden found that noise annoyance among university students in laboratory environment was positively associated with self-reported headaches [33]. Another study from Sweden found that annoyance from road traffic noise among residents was related to tiredness [34]. Moreover, one Swedish study suggested that exposing to sound levels from road traffic ranging from 45 to 68 dBA at the most exposed side may induce tiredness among residents [35]. Some studies indicated that traffic noise exposure or traffic noise induced annoyance were associated with mental stress or mental disorders among residents [34–37]. The results in our study is in agreement with previous studies on noise annoyance from outdoor sources (mainly outdoor traffic) and stress symptoms/metal disorders. Our study indicate that noise disturbance from indoor sources (e.g. neighbours, installations and ventilation/fans/heat pumps) can affect these types of symptoms, which is a new finding.

### Personal factors

#### Age

Younger participants reported more noise disturbance as compared to older participants (> 65 y). Moreover, we found that medical symptoms were more prevalent among younger participants as compared to older participants (> 65 y). Few studies have investigated age in association with noise annoyance. One study from India found that middle-aged subjects were more annoyed by road traffic noise as compared to younger and older subjects [38]. One study from France indicated that older participants were more likely to be highly annoyed by aircraft noise [11]. One explanation of our results is that younger participants can have higher demands on their home environment. Another explanation can be that the elderly have higher prevalence of hearing loss.

#### Gender

We found that females reported more voice/radio/TV/music/similar sounds as compared to males. No gender difference was found regarding other noise complaints. We found no previous studies investigating gender in relation to noise annoyance from indoor sources.

#### Smoking

Current smokers reported more noise disturbance and more medical symptoms (tiredness and headache) as compared to non-smokers in our study. Smoking is usually considered to be a socioeconomic factor. Smokers can have a lower socioeconomic status and can be living in buildings with poorer condition as compared to non-smokers. The sound or noise condition at home can be different between smokers and non-smokers due to different life style. In addition, one recent review suggested that there can be physiological interaction between noise exposure and smoking for auditory and non-auditory health effect [39].

#### Living period in the current apartment

A longer living time in the current apartment (more than 5 yrs) was associated with more reports of noise from neighbours. People with a longer living period in the current home may have less tolerance to noise and can be more sensitive to noise from neighbourhood.

### Climate zone

Living in a warmer climate was related to more noise disturbance from amusement centre in the property and noise disturbance from stairwell/elevators inside the building in our study. We found no previous studies on climate and noise annoyance. Warm climate can indicate more outdoor activities, especially in warmer seasons. People living in warmer climate are more used to open windows, spending time on balconies/outside the building in warmer seasons. Amusement centre can have open windows and have more people staying outside of the centre in warmer seasons. The reason why subjects living in a warmer zone reported more noise disturbance from stairwell/elevators is unclear. One explanation could be that the use of stairwell/elevators was more frequent due to more common outdoor activities among occupants living in warmer climate zones.

### Urbanization

We found that increased municipality population density was associated with more noise disturbance from different indoor and outdoor sources linked to various noise sources. A higher municipality population density indicates a higher degree of urbanization linked to different noise sources.

### Building related factors

#### Ownership

Renting the apartment was associated with noise disturbance from different indoor and outdoor sources in our study. Previous Swedish study indicated that a large amount of multi-family buildings are owned by the community and used for renting [40]. Multi-family buildings constructed from 1961 to 1975 in Sweden are mostly rented and prioritized for renovation in nowadays. Those buildings often have poor sound insulation due to thinner walls, no triple glass windows and poorer insulation in floors. Moreover, people living in rented building areas are more likely to have less social control as compared to those living in self-owned building areas. Participants living in rented apartments can have a low socioeconomic status. However, we found no association between crowdedness (one indicator of socioeconomic status) and noise disturbance. It is more likely that participants living in rented apartments were living in buildings with poorer acoustic conditions (poor sound insulation, more noise from outside) and had worse social control (less respect between tenants) than those living in self-owned apartments.

#### Building construction year

We found that construction year from 1961 to 1985 was more likely to be associated with noise disturbance in general. Living in newer buildings (constructed from 1986 to 2005) was related to less reporting of noise disturbance. We have found previously that subjects living in multi-family buildings built during 1961–1985 in Sweden had more respiratory illnesses [27]. Multi-family buildings from 1961 to 1975 in Sweden were mostly poor quality high-rise buildings rented by poorer people. They were constructed with thinner walls, poorer insulation in floors and only double glass windows (no triple glass windows). In Sweden, apartment buildings from 1976 to 1985 often have thinner walls with less sound insulation and double glass windows. Newer apartment buildings have better insulation and tripple glazing. Thus, poor sound insulation can be the main reason to noise disturbance found in our study.

#### Mechanical ventilation and window opening

Having mechanical ventilation was associated more noise disturbance from ventilation/fans inside the building and ventilation/fans/heat pumps outside the building. Noise from mechanical ventilation system can disturbs, especially in buildings with poor sound insulation. Reducing noise impact on residents when installing a mechanical ventilation system can be important.

Daily window opening was related to less noise disturbance from ventilation/fans/heat pumps. People with the habit of frequently opening windows can be less sensitive to constant noise from ventilation systems.

### The mediation effects of noise disturbance

The mediation analyses showed that the associations between selected building factors (older buildings, renting the apartment and lack of mechanical ventilation) and medical symptoms were partly mediated by noise disturbance in general at home.

### Strengths and limitations

#### Strengths

The present study is one of a few studies on noise disturbance that covered a sample of a whole country. All subjects (≥ 18 years) living in multi-family buildings were included in the present study, with no prior information on their health status. There was no major difference in participation rate between different municipalities, between those being born in Sweden and foreign-born persons, and between Swedish citizens or non-Swedish citizens. Most of the differences in participation rate were small [27]. Some factors discussed such as temperature zone and population density were not obtained from the self-administered questionnaire, which reduces potential reporting bias.

#### Limitations

The response rate was not very high in our study (46%). The participation rate was higher among elderly and married persons, but slightly lower in larger cities, suburban municipalities and in older buildings [27]. The comparison between participants and non-participants was performed by SCB Sweden, by linking personal identity from the questionnaires to related register data. Due to the ethical aspect, we could not access to the register data. Only the questionnaire data on responders were available for us. There were no data on other chronic diseases except for asthma and allergies in the BETSI questionnaire. There were no questions on the time when noise disturbance occurs, acute stress events and noise sensitivity in the questionnaire. Information bias can occur in questionnaire surveys. Subjects may overestimate or underestimate environment risk factors as well as medical symptoms. We found some associations between specific home environment risk factors and noise disturbance, not a similar association for all risk factors. Thus, our findings are less likely to be seriously biased by selection or information bias.

## Conclusions

Noise disturbance from different indoor and outdoor sources can be common among occupants living in multi-family buildings in Sweden. Noise disturbance can be related to tiredness, headache and difficulty concentrating. Younger participants and smokers may report more noise disturbance. Living in a warmer climate, living in areas with higher municipality population density (indicating higher degree of urbanization), living in rented apartments and living in buildings constructed from 1961 to 1985 can be associated with more noise disturbance from different indoor and outdoor sources. The associations between building risk factors (older buildings, renting the apartment and lack of mechanical ventilation) and medical symptoms can be partly mediated by noise disturbance.

## Supplementary Information

**Additional file 1.** The associations between home environment factors and noise disturbance from indoor and outdoor sources. The associations were described in odds ratios with 95% confidence interval

## Data Availability

The datasets used and/or analyzed during the current study are available from the corresponding author on reasonable request.

## Abbreviations

BETSI: Building Energy, Technical Status and Indoor Environment
CI: confidence interval
DALY: disability-adjusted life-years
dBA: A-weighted decibels
OR: odds ratios
SCB: Statistics Sweden (In Swedish: Statistiska centralbyrån)
WHO: World Health Organization
