# Correction: Home environment and noise disturbance in a national sample of multi-family buildings in Sweden-associations with medical symptoms

**DOI:** 10.1186/s12889-022-13437-w

**Published:** 2022-06-06

**Authors:** Juan Wang, Dan Norbäck

**Affiliations:** Department of Medical Sciences, Occupational and Environmental Medicine, Uppsala University, SE-751 85 Uppsala, Sweden

**Correction: BMC Public Health 21, 1989 (2021)**

**https://doi.org/10.1186/s12889-021-12069-w**

In the original [1] publication of this paper figures 2 and 4 were swapped, the captions and citations were correct. The article has been updated with the correct figures.
