# Supplementary material for: Home environment and noise disturbance in a national sample of multi-family buildings in Sweden-associations with medical symptoms
Source: BMC Public Health. 2021 Nov 3;21:1989. doi: 10.1186/s12889-021-12069-w (PMC8565173; doi:10.1186/s12889-021-12069-w)
Supplement: Supplementary file 1 — Additional file 1. The associations between home environment factors and noise disturbance from indoor and outdoor sources. The associations were described in odds ratios with 95% confidence interval [file 12889_2021_12069_MOESM1_ESM.docx]

**Additional file 1**

Table S1. Associations between home environment factors and noise disturbance in general at home OR(95%CI) ^a^.

| Home environment factors |  | Median (min, max) | Total n=5775 (%) | Noise disturbance in general at home | p |
| --- | --- | --- | --- | --- | --- |
| Temperature zone ^b^ |  | 3(1, 4) |  | 1.10(0.93,1.30) | 0.288 |
| Municipality population density ^c^ | Total | 1179(0.29, 4732) |  | 0.85(0.32,2.28) | 0.745 |
|  | Quartile 1 | 27.7(0.29,64.7) |  | - | - |
|  | Quartile 2 | 304(75.5,1179) |  | - | - |
|  | Quartile 3 | 1970(1448,3709) |  | - | - |
|  | Quartile 4 | 4732(4732,4732) |  | - | - |
| Crowdedness ^d^ |  | 2.30(0.51,27.6) |  | 1.06(0.999,1.12) | 0.055 |
| Total number of persons | Continuous |  |  | 1.02(0.94,1.10) | 0.687 |
| Total number of persons | 1 |  | 41.6 | 1.00 |  |
|  | 2 |  | 40.1 | 0.86(0.71,1.04) | 0.120 |
|  | 3 |  | 9.8 | 1.03(0.78,1.36) | 0.836 |
|  | 4 or more |  | 8.5 | 1.02(0.76,1.38) | 0.878 |
| Time living in current apartment | ≤ 5 years |  | 47.4 | 1.00 |  |
|  | > 5 years |  | 52.6 | 1.30(1.09,1.54) | **0.004** |
| Construction year | -1960 |  | 12.5 | 1.00 |  |
|  | 1961-1975 |  | 33.0 | 1.35(1.04,1.74) | **0.022** |
|  | 1976-1985 |  | 17.9 | 0.93(0.69,1.26) | 0.658 |
|  | 1986-1995 |  | 16.0 | 0.67(0.48,0.92) | **0.015** |
|  | 1996-2005 |  | 20.7 | 0.39(0.28,0.55) | **<0.001** |
| Ownership | Self-owned |  | 49.3 | 1.00 |  |
|  | Renting |  | 50.7 | 2.70(2.24,3.26) | **<0.001** |
| Location of the apartment | Ground floor/basement |  | 18.2 | 1.00 |  |
|  | Above ground floor |  | 81.8 | 1.31(1.04,1.66) | **0.021** |
| Any mechanical ventilation | Yes |  | 54.6 | 0.65(0.54,0.78) | **<0.001** |
| Bathroom fan | Yes |  | 14.6 | 0.83(0.64,1.06) | 0.139 |
| Window opening frequency | Less often |  | 26.1 | 1.00 |  |
|  | Everyday |  | 73.9 | 1.09(0.90,1.32) | 0.370 |

Bold values indicate p<0.05.

^a^ Two-level logistic regression models (individual, municipality) by including only one home environment factor in each model. The odds ratios were adjusted for gender, age and smoking.

^b^ The ORs were expressed per 1 unit increase for temperature zone.

^c^ The ORs were expressed per 1000 increase for municipality population density (number of persons per km^2^, person/km^2^).

^d^ The ORs were expressed per 1 unit increase for crowdedness (person/100m^2^).

Table S2. The correlation between noise disturbance factors (Spearman correlation coefficients).

| Noise disturbance | q1 | q2 | q3 | q4 | q5 | q6 | q7 | q8 | q9 | q10 | q11 |
| --- | --- | --- | --- | --- | --- | --- | --- | --- | --- | --- | --- |
| Noise disturbance in general at home (q1) | 1.00 |  |  |  |  |  |  |  |  |  |  |
| Noise from inside: lines and pipes (q2) | 0.28 | 1.00 |  |  |  |  |  |  |  |  |  |
| Noise from inside: ventilation/fans inside (q3) | 0.23 | 0.28 | 1.00 |  |  |  |  |  |  |  |  |
| Noise from inside: voice, radio, TV, music or similar sounds from neighbors (q4) | 0.47 | 0.31 | 0.20 | 1.00 |  |  |  |  |  |  |  |
| Noise from inside: scraping sound, footsteps, thumping or similar sounds from neighbors (q5) | 0.44 | 0.31 | 0.16 | 0.57 | 1.00 |  |  |  |  |  |  |
| Noise from inside: amusement centre in the property (q6) | 0.19 | 0.22 | 0.19 | 0.21 | 0.22 | 1.00 |  |  |  |  |  |
| Noise from inside: stairwell, elevators (q7) | 0.35 | 0.26 | 0.16 | 0.38 | 0.41 | 0.16 | 1.00 |  |  |  |  |
| Noise from outside: ventilation/fans/warm pumps (q8) | 0.16 | 0.20 | 0.48 | 0.11 | 0.11 | 0.20 | 0.16 | 1.00 |  |  |  |
| Noise from outside: road traffic (q9) | 0.28 | 0.16 | 0.15 | 0.18 | 0.21 | 0.13 | 0.21 | 0.15 | 1.00 |  |  |
| Noise from outside: train traffic (q10) | 0.09 | 0.09 | 0.08 | 0.08 | 0.10 | 0.11 | 0.10 | 0.16 | 0.23 | 1.00 |  |
| Noise from outside: flight traffic (q11) | 0.10 | 0.10 | 0.13 | 0.10 | 0.12 | 0.11 | 0.12 | 0.13 | 0.19 | 0.23 | 1.00 |

Table S3. The correlation between home environment factors (Spearman correlation coefficients).

|  | q1 | q2 | q3 | q4 | q5 | q6 | q7 | q8 | q9 | q10 |
| --- | --- | --- | --- | --- | --- | --- | --- | --- | --- | --- |
| Temperature zone (q1) | 1.00 |  |  |  |  |  |  |  |  |  |
| Municipality population density (q2) | 0.23 | 1.00 |  |  |  |  |  |  |  |  |
| Crowdedness (q3) | 0.03 | 0.18 | 1.00 |  |  |  |  |  |  |  |
| Time living in current apartment (q4) | 0.05 | 0.04 | -0.06 | 1.00 |  |  |  |  |  |  |
| Construction year (q5) | -0.10 | -0.04 | -0.02 | -0.23 | 1.00 |  |  |  |  |  |
| Ownership (q6) | -0.01 | -0.16 | 0.13 | -0.06 | -0.11 | 1.00 |  |  |  |  |
| Location of the apartment (q7) | 0.04 | 0.14 | -0.00 | 0.02 | -0.04 | -0.03 | 1.00 |  |  |  |
| Any mechanical ventilation (q8) | -0.06 | -0.03 | -0.06 | -0.04 | 0.30 | -0.12 | 0.00 | 1.00 |  |  |
| Bathroom fan (q9) | 0.04 | -0.05 | -0.05 | -0.01 | -0.00 | 0.01 | -0.01 | -0.10 | 1.00 |  |
| Window opening frequency (q10) | 0.07 | -0.04 | -0.14 | 0.03 | 0.007 | -0.03 | 0.04 | 0.05 | 0.04 | 1.00 |

Table S4. Associations between home environment factors and noise disturbance from specific indoor sources OR(95%CI) ^a^.

| Home environment factors |  | Lines and pipes | p | Ventilation/fans inside | p | Voice, radio, TV, music or similar sounds from neighbors | p |
| --- | --- | --- | --- | --- | --- | --- | --- |
| Temperature zone ^b^ |  | 1.09(0.93,1.29) | 0.276 | 0.95(0.78,1.16) | 0.638 | 0.93(0.76,1.13) | 0.451 |
| Municipality population density ^c^ |  | 1.05(0.98,1.12) | 0.177 | 1.02(0.93,1.13) | 0.662 | 0.98(0.86,1.12) | 0.782 |
| Time living in current apartment | ≤ 5 years | 1.00 |  | 1.00 |  | 1.00 |  |
|  | > 5 years | 1.12(0.91,1.38) | 0.275 | 0.92(0.73,1.14) | 0.436 | 1.30(1.09,1.55) | **0.003** |
| Construction year | -1960 | 1.00 |  | 1.00 |  | 1.00 |  |
|  | 1961-1975 | 2.25(1.58,3.19) | **<0.001** | 1.49(0.99,2.24) | 0.055 | 1.47(1.14,1.89) | **0.003** |
|  | 1976-1985 | 1.35(0.90,2.01) | 0.147 | 2.38(1.57,3.63) | **<0.001** | 0.83(0.61,1.12) | 0.221 |
|  | 1986-1995 | 1.46(0.97,2.19) | 0.071 | 2.27(1.48,3.49) | **<0.001** | 0.74(0.54,1.02) | 0.066 |
|  | 1996-2005 | 1.00(0.67,1.51) | 0.991 | 0.92(0.58,1.46) | 0.720 | 0.36(0.25,0.50) | **<0.001** |
| Ownership | Self-owned | 1.00 |  | 1.00 |  | 1.00 |  |
|  | Renting | 2.11(1.70,2.62) | **<0.001** | 1.64(1.31,2.05) | **<0.001** | 2.31(1.93,2.78) | **<0.001** |
| Location of the apartment | Ground floor/basement | 1.00 |  | 1.00 |  | 1.00 |  |
|  | Above ground floor | 1.27(0.96,1.68) | 0.094 | 0.98(0.74,1.29) | 0.871 | 1.35(1.07,1.71) | **0.011** |
| Any mechanical ventilation | Yes | 0.73(0.59,0.92) | **0.006** | 1.26(0.99,1.61) | 0.063 | 0.72(0.60,0.88) | **0.001** |
| Bathroom fan | Yes | 1.11(0.84,1.47) | 0.448 | 0.91(0.66,1.25) | 0.542 | 1.18(0.94,1.49) | 0.153 |
| Window opening frequency | Less often | 1.00 |  | 1.00 |  | 1.00 |  |
|  | Everyday | 1.05(0.84,1.31) | 0.681 | 0.74(0.59,0.93) | **0.011** | 0.97(0.80,1.16) | 0.720 |

Bold values indicate p<0.05.

^a^ Two-level logistic regression models (individual, municipality) by including only one home environment factor in each model. The odds ratios were adjusted for gender, age and smoking.

^b^ The ORs were expressed per 1 unit increase for temperature zone.

^c^ The ORs were expressed per 1000 increase for municipality population density (number of persons per km^2^).

Table S5. Associations between home environment factors and noise disturbance from specific indoor sources OR(95%CI) ^a^.

| Home environment factors |  | Scraping sound, footsteps, thumping or similar sounds from neighbours | p | Amusement centre in the property | p | Stairwell, elevators | p |
| --- | --- | --- | --- | --- | --- | --- | --- |
| Temperature zone ^b^ |  | 0.99(0.81,1.20) | 0.911 | 1.98(1.15,3.40) | **0.014** | 1.17(0.96,1.42) | 0.126 |
| Municipality population density ^c^ |  | 1.03(0.91,1.16) | 0.690 | 1.23(0.92,1.66) | 0.166 | 0.96(0.85,1.07) | 0.449 |
| Time living in current apartment | ≤ 5 years | 1.00 |  | 1.00 |  | 1.00 |  |
|  | > 5 years | 1.38(1.17,1.62) | **<0.001** | 1.20(0.81,1.79) | 0.362 | 1.19(0.98,1.45) | 0.081 |
| Construction year | -1960 | 1.00 |  | 1.00 |  | 1.00 |  |
|  | 1961-1975 | 1.37(1.08,1.73) | **0.008** | 2.75(1.43,5.29) | **0.003** | 1.29(0.97,1.72) | 0.079 |
|  | 1976-1985 | 0.76(0.57,1.00) | 0.050 | 0.91(0.39,2.09) | 0.820 | 0.87(0.62,1.23) | 0.430 |
|  | 1986-1995 | 0.79(0.59,1.05) | 0.102 | 1.54(0.70,3.39) | 0.289 | 0.80(0.56,1.14) | 0.210 |
|  | 1996-2005 | 0.50(0.37,0.66) | **<0.001** | 0.62(0.26,1.48) | 0.283 | 0.52(0.36,0.78) | **<0.001** |
| Ownership | Self-owned | 1.00 |  | 1.00 |  | 1.00 |  |
|  | Renting | 1.91(1.62,2.24) | **<0.001** | 2.76(1.78,4.28) | **<0.001** | 2.33(1.89,2.86) | **<0.001** |
| Location of the apartment | Ground floor/basement | 1.00 |  | 1.00 |  | 1.00 |  |
|  | Above ground floor | 1.20(0.98,1.48) | 0.080 | 1.18(0.70,2.01) | 0.537 | 0.89(0.70,1.14) | 0.349 |
| Any mechanical ventilation | Yes | 0.85(0.72,1.01) | 0.066 | 0.76(0.49,1.17) | 0.209 | 0.72(0.58,0.88) | **0.002** |
| Bathroom fan | Yes | 1.02(0.82,1.27) | 0.857 | 1.92(1.22,3.02) | **0.005** | 1.13(0.87,1.46) | 0.361 |
| Window opening frequency | Less often | 1.00 |  | 1.00 |  | 1.00 |  |
|  | Everyday | 0.98(0.83,1.16) | 0.803 | 0.81(0.53,1.21) | 0.295 | 0.90(0.73,1.11) | 0.340 |

Bold values indicate p<0.05.

^a^ Two-level logistic regression models (individual, municipality) by including only one home environment factor in each model. The odds ratios were adjusted for gender, age and smoking.

^b^ The ORs were expressed per 1 unit increase for temperature zone.

^c^ The ORs were expressed per 1000 increase for municipality population density (number of persons per km^2^).

Table S6. Associations between home environment factors and noise disturbance from specific outdoor sources OR(95%CI)^a^.

| Home environment factors |  | Ventilation/fans/warm pumps | p | Road traffic | p | Train traffic | p | Flight traffic | p | Any severe traffic noise effect (often vs. less or never) | p |
| --- | --- | --- | --- | --- | --- | --- | --- | --- | --- | --- | --- |
| Temperature zone ^b^ |  | 1.25(0.99,1.58) | 0.066 | 1.08(0.88,1.33) | 0.435 | 1.12(0.60,2.07) | 0.722 | 1.23 (0.45,3.33) | 0.684 | 1.32(0.99,1.75) | 0.058 |
| Municipality population density ^c^ |  | 1.09(1.02,1.17) | **0.009** | 1.02(0.89,1.17) | 0.750 | 1.36(0.95,1.96) | 0.097 | 2.00(1.11,3.61) | **0.021** | 1.08(0.90,1.28) | 0.407 |
| Time living in current apartment | ≤ 5 years | 1.00 |  | 1.00 |  | 1.00 |  | 1.00 |  | 1.00 |  |
|  | > 5 years | 1.23(0.93,1.61) | 0.145 | 0.99(0.85,1.16) | 0.906 | 1.08(0.78,1.51) | 0.645 | 1.30(0.94,1.80) | 0.113 | 1.13(0.91,1.41) | 0.269 |
| Construction year | -1960 | 1.00 |  | 1.00 |  | 1.00 |  | 1.00 |  | 1.00 |  |
|  | 1961-1975 | 1.84(1.11,3.06) | **0.019** | 0.95(0.76,1.20) | 0.692 | 3.65(1.73,7.68) | **0.001** | 1.76(1.02,3.02) | **0.041** | 1.04(0.74,1.46) | 0.831 |
|  | 1976-1985 | 2.60(1.53,4.41) | **<0.001** | 0.80(0.61,1.04) | 0.095 | 3.13(1.42,6.87) | **0.005** | 2.42(1.38,4.26) | **0.002** | 1.13(0.78,1.65) | 0.524 |
|  | 1986-1995 | 2.11(1.22,3.65) | **0.008** | 0.82(0.63,1.08) | 0.155 | 2.21(0.96,5.12) | 0.063 | 1.43(0.79,2.60) | 0.240 | 0.98(0.66,1.45) | 0.904 |
|  | 1996-2005 | 0.91(0.50,1.64) | 0.748 | 0.68(0.52,0.88) | **0.003** | 3.26(1.51,7.04) | **0.003** | 0.37(0.17,0.79) | **0.011** | 0.62(0.42,0.93) | **0.022** |
| Ownership | Self-owned | 1.00 |  | 1.00 |  | 1.00 |  | 1.00 |  | 1.00 |  |
|  | Renting | 1.74(1.32,2.30) | **<0.001** | 1.85(1.58,2.16) | **<0.001** | 2.34(1.65,3.32) | **<0.001** | 1.64(1.19,2.25) | **0.002** | 1.85(1.47,2.33) | **<0.001** |
| Location of the apartment | Ground floor/basement | 1.00 |  | 1.00 |  | 1.00 |  | 1.00 |  | 1.00 |  |
|  | Above ground floor | 1.06(0.74,1.50) | 0.762 | 1.06(0.87,1.29) | 0.563 | 0.91(0.59,1.39) | 0.650 | 0.94(0.61,1.44) | 0.773 | 1.35(0.996,1.83) | 0.053 |
| Any mechanical ventilation | Yes | 1.35(1.004,1.82) | **0.047** | 0.84(0.72,0.996) | **0.045** | 0.91(0.64,1.29) | 0.587 | 0.96(0.68,1.35) | 0.814 | 0.80(0.63,1.02) | 0.070 |
| Bathroom fan | Yes | 0.93(0.63,1.37) | 0.703 | 1.02(0.83,1.27) | 0.826 | 0.75(0.45,1.26) | 0.274 | 1.16(0.74,1.84) | 0.518 | 1.12(0.83,1.52) | 0.450 |
| Window opening frequency | Less often | 1.00 |  | 1.00 |  | 1.00 |  | 1.00 |  | 1.00 |  |
|  | Everyday | 0.76(0.57,1.01) | 0.059 | 1.14(0.96,1.35) | 0.136 | 1.14(0.79,1.63) | 0.481 | 1.65(1.13,2.42) | **0.010** | 1.25(0.97,1.60) | 0.084 |

Bold values indicate p<0.05.

^a^ Two-level logistic regression models (individual, municipality) by including only one home environment factor in each model. The odds ratios were adjusted for gender, age and smoking.

^b^ The ORs were expressed per 1 unit increase for temperature zone.

^c^ The ORs were expressed per 1000 increase for municipality population density (number of persons per km^2^).

Table S7. Associations between home environment factors and medical symptoms OR(95%CI) ^a^.

| Home environment factors |  | Any weekly symptom | p | Weekly tiredness | p | Weekly headache | p | Weekly difficulty concentrating | p |
| --- | --- | --- | --- | --- | --- | --- | --- | --- | --- |
| Temperature zone ^b^ |  | 1.01(0.90,1.14) | 0.840 | 1.02(0.93,1.13) | 0.606 | 1.03(0.89,1.19) | 0.682 | 1.03(0.86,1.23) | 0.741 |
| Municipality population density ^c^ | Total | 0.98(0.58,1.68) | 0.954 | 1.06(0.74,1.51) | 0.759 | 1.05(0.61,1.81) | 0.851 | 0.88(0.45,1.73) | 0.716 |
|  | Quartile 1 |  |  |  |  |  |  |  |  |
|  | Quartile 2 |  |  |  |  |  |  |  |  |
|  | Quartile 3 |  |  |  |  |  |  |  |  |
|  | Quartile 4 |  |  |  |  |  |  |  |  |
| Crowdedness ^d^ |  | 1.01(0.96,1.06) | 0.613 | 1.00(0.95,1.06) | 0.875 | 1.06(0.99,1.14) | 0.114 | 1.04(0.95,1.14) | 0.405 |
| Total number of persons | Continuous | 0.99(0.94,1.06) | 0.873 | 0.98(0.92,1.04) | 0.512 | 1.11(1.02,1.21) | **0.021** | 1.02(0.91,1.15) | 0.722 |
| Total number of persons | 1 | 1.00 |  | 1.00 |  | 1.00 |  |  |  |
|  | 2 | 0.82(0.71,0.95) | **0.009** | 0.82(0.71,0.95) | **0.010** | 0.98(0.77,1.24) | 0.878 | 0.64(0.48,0.86) | **0.003** |
|  | 3 | 0.76(0.61,0.97) | **0.025** | 0.77(0.61,0.98) | **0.036** | 1.16(0.82,1.63) | 0.414 | 0.65(0.41,1.05) | 0.077 |
|  | 4 or more | 1.15(0.91,1.45) | 0.238 | 1.11(0.87,1.41) | 0.393 | 1.48(1.06,2.08) | **0.022** | 1.19(0.79,1.78) | 0.407 |
| Time living in current apartment | ≤ 5 years | 1.00 |  | 1.00 |  | 1.00 |  |  |  |
|  | > 5 years | 0.88(0.77,1.01) | 0.078 | 0.85(0.74,0.98) | **0.023** | 1.03(0.83,1.29) | 0.773 | 0.95(0.73,1.23) | 0.689 |
| Construction year | -1960 | 1.00 |  | 1.00 |  | 1.00 |  | 1.00 |  |
|  | 1961-1975 | 0.98(0.79,1.20) | 0.821 | 0.91(0.74,1.13) | 0.406 | 1.76(1.24,2.51) | **0.002** | 1.33(0.89,2.00) | 0.166 |
|  | 1976-1985 | 1.02(0.81,1.29) | 0.879 | 0.99(0.78,1.26) | 0.931 | 1.60(1.08,2.36) | **0.020** | 1.21(0.77,1.90) | 0.416 |
|  | 1986-1995 | 0.98(0.77,1.25) | 0.886 | 1.00(0.78,1.28) | 0.998 | 1.39(0.93,2.10) | 0.111 | 1.18(0.74,1.89) | 0.479 |
|  | 1996-2005 | 0.72(0.57,0.91) | **0.005** | 0.76(0.60,0.97) | **0.024** | 0.84(0.55,1.29) | 0.426 | 0.50(0.29,0.85) | **0.010** |
| Any children | Yes | 1.13(0.93,1.37) | 0.218 | 1.15(0.94,1.40) | 0.171 | 1.40(1.07,1.85) | **0.015** | 0.98(0.67,1.42) | 0.906 |
| Ownership | Self-owned | 1.00 |  |  |  |  |  |  |  |
|  | Renting | 1.44(1.26,1.65) | **<0.001** | 1.33(1.16,1.52) | **<0.001** | 1.94(1.56,2.41) | **<0.001** | 1.70(1.30,2.22) | **<0.001** |
| Location of the apartment | Ground floor/basement | 1.00 |  |  |  |  |  |  |  |
|  | Above ground floor | 1.00(0.84,1.19) | 0.985 | 1.02(0.85,1.21) | 0.863 | 1.06(0.81,1.38) | 0.697 | 1.22(0.86,1.73) | 0.275 |
| Any mechanical ventilation | Yes | 0.83(0.72,0.96) | **0.014** | 0.89(0.77,1.04) | 0.136 | 0.75(0.59,0.95) | **0.015** | 0.78(0.59,1.03) | 0.086 |
| Bathroom fan | Yes | 1.04(0.86,1.25) | 0.707 | 0.93(0.77,1.14) | 0.496 | 1.03(0.77,1.38) | 0.839 | 1.08(0.75,1.54) | 0.690 |
| Window opening frequency | Less often | 1.00 |  | 1.00 |  | 1.00 |  | 1.00 |  |
|  | Everyday | 0.96(0.83,1.11) | 0.567 | 0.96(0.83,1.12) | 0.604 | 1.06(0.84,1.34) | 0.609 | 1.18(0.88,1.57) | 0.278 |

Bold values indicate p<0.05.

^a^ Two-level logistic regression models (individual, municipality) by including only one home environment factor in each model. The odds ratios were adjusted for gender, age and smoking.

^b^ The ORs were expressed per 1 unit increase for temperature zone.

^c^ The ORs were expressed per 1000 increase for municipality population density (number of persons per km^2^, person/km^2^).

^d^ The ORs were expressed per 1 unit increase for crowdedness (person/100m^2^).
